# Traditional herbal medicine for opioid-induced constipation in patients with cancer: a systematic review and meta-analysis of randomized controlled trials

**DOI:** 10.3389/fphar.2025.1716974

**Published:** 2026-01-16

**Authors:** Su Hyeon Lee, Hayun Jin, Eun Hye Kim, Seong Woo Yoon

**Affiliations:** 1 Department of Korean Internal Medicine, Kyung Hee University Hospital at Gangdong, Seoul, Republic of Korea; 2 Department of Internal Medicine, College of Korean Medicine, Gachon University, Gyeonggi-Do, Republic of Korea

**Keywords:** cancer, meta-analysis, opioid-induced constipation, systematic review, traditional herbal medicine

## Abstract

**Introduction:**

This systematic review and meta-analysis aimed to evaluate the efficacy and safety of traditional herbal medicine (THM) in improving opioid-induced constipation (OIC) in patients with cancer.

**Methods:**

To identify randomized controlled trials (RCTs) evaluating orally administered THM for OIC in patients with cancer, a comprehensive search of seven databases was conducted from inception to 29 August 2024. The primary outcome was improvement in OIC, which was assessed using the total effective rate (TER). Secondary outcomes included stool form, difficulty of defecation, defecation time, and the Karnofsky performance scale (KPS). The methodological quality of the included studies was assessed using the Cochrane Risk of Bias tool, and the certainty of evidence was evaluated according to the Grading of Recommendations Assessment, Development, and Evaluation method.

**Results:**

In total, 21 RCTs involving 2,108 patients were included. Compared to conventional medicine, THM significantly improved OIC as measured by TER [risk ratio (RR) 1.21, 95% confidence intervals (CIs) 1.14–1.25], with high certainty. THM showed a significant improvement in stool form [mean difference (MD) −0.16, 95% CIs −0.43–0.10; very low certainty], difficulty of defecation [MD -0.31, 95% CIs −0.49 to −0.13; low certainty], defecation time [MD -0.28, 95% CIs −0.45 to −0.10; moderate certainty], and KPS measured by mean changes in scores [MD 6.76, 95% CIs 4.32–9.20; low certainty]. Adverse events were mainly gastrointestinal symptoms such as diarrhea, nausea, and abdominal pain, but such events were not serious.

**Conclusion:**

The findings of this systematic review indicate that THM may be considered a safe and potentially alternative option for improving OIC in patients with cancer. However, more robust and high-quality RCTs are required to strengthen this evidence.

**Systematic Review Registration:**

https://www.crd.york.ac.uk/prospero, Identifier: CRD42024557773.

## 1 Introduction

Pain is one of the most burdensome symptoms experienced by patients with cancer. Although the prevalence and severity of pain in patients with cancer have declined over the past decade, the prevalence of pain remains high, especially in patients with advanced, metastatic, and terminal cancer (Snijders et al., 2023). Cancer pain has a multidimensional etiology, which has a significant impact on the overall quality of life of patients with cancer by its influence on physical, psychological, and spiritual domains (Ahmedzai, 1995). The American Society of Clinical Oncology (ASCO) guidelines recommend the use of opioids such as codeine, hydrocodone, and oxycodone for the treatment of moderate-to-severe pain related to cancer or active treatment in adult cancer patients (Paice et al., 2023).

Although cancer pain management has mostly relied on opioids, they commonly induce side effects such as nausea, vomiting, sedation, hallucination, respiratory depression, and constipation (Mestdagh et al., 2023). Opioid-induced constipation (OIC) is defined as a change from baseline bowel habits when initiating opioid therapy that is characterized by any of the following: reduced bowel movement frequency, development or worsening of straining to pass bowel movements, a sense of incomplete rectal evacuation, or harder stool consistency (Camilleri et al., 2014). OIC is the most commonly reported side effect among patients receiving opioids. In patients with cancer pain, the prevalence of OIC, as indicated by the use of laxatives, a surrogate parameter, was reported to be 94% (Sykes, 1998). OIC compromises patient satisfaction with analgesic treatment and significantly impairs quality of life (AlMouaalamy, 2021).

Laxatives are recommended as first-line treatment for OIC, with peripherally acting μ-opioid receptor antagonists (PAMORAs) reserved for laxative-refractory cases; however, laxatives do not directly target the μ-opioid receptor-mediated pathophysiology of OIC, nor do they avoid frequently reported gastrointestinal adverse effects such as bloating, flatulence, and fecal urgency (Emmanuel et al., 2017; Crockett et al., 2019). Recent reviews have shown that the long-term or repeated use of simulant laxatives may induce structural alterations in the colonic epithelium, raising concerns about their safety when used chronically (Whorwell et al., 2024). Therefore, alternative therapeutic options need to be identified.

Traditional herbal medicine (THM) is widely used to treat various subtypes of constipation, including functional and postpartum constipation (Zhai et al., 2019; Lyu et al., 2022), and recent randomized controlled trials (RCTs) have investigated its use for OIC in patients with cancer (Chen et al., 2019; Wei and Du, 2020; Liu et al., 2022). However, no meta-analyses have specifically focused on the use of THM in this area, and the evidence remains limited for patients with cancer pain. In parallel, a recent systematic review has highlighted beneficial effects of rehabilitation, osteopathy and acupuncture on OIC in oncological patients, but it did not evaluate THM as a therapeutic option (Chiaramonte et al., 2023). Therefore, this systematic review and meta-analysis of RCTs aimed to assess the efficacy and safety of THM in patients who develop constipation following the use of opioid analgesics for cancer pain.

## 2 Methods

This systematic review and meta-analysis were performed according to the Preferred Reporting Items for Systematic Reviews and Meta-Analyses (PRISMA) checklist (Page et al., 2021). The protocol for this review was registered in the International Prospective Register of Systematic Review (PROSPERO) with the registration number CRD42024557773.

### 2.1 Data source and search strategy

PubMed, Cochrane Library, Embase, China National Knowledge Infrastructure (CNKI), Korean databases (KMBASE, KISS, NDSL), and Japanese database (CiNii) were searched for articles published until 29 August 2024. The search terms included cancer, neoplasm, opioid-induced constipation, traditional herbal medicine, and traditional Chinese medicine. The detailed search strategies used for each database are provided in Supplementary Material S1. Language restrictions were not imposed. Two reviewers independently screened the titles and abstracts to select studies that met the eligibility criteria and reviewed the full texts to determine whether the studies should be included. In cases of disagreement, a third researcher made the final decision. No ethical approval was required because all research materials were previously published.

### 2.2 Study selection

Two investigators independently screened the titles and abstracts of all retrieved studies for eligibility and subsequently assessed the full text of the relevant articles. Clinical RCTs were eligible for inclusion according to the PICOS criteria as follows.

1. Participants (P): Patients who developed constipation after receiving opioid analgesics for cancer pain, regardless of age, sex, ethnicity, or cancer type.
2. Interventions (I): Experimental groups receiving orally administered THM alone as the experimental intervention, regardless of dosage or duration.
3. Comparisons (C): Control groups receiving conventional medicine (CM), placebo, or no treatment (e.g., waiting-list group). CM was defined as treatments recommended by clinical guidelines (National Comprehensive Cancer Network, 2025) (e.g., stimulant and osmotic laxatives) as well as other commonly used agents in clinical practice such as prokinetics.
4. Outcomes (O): Outcome measures including the total effective rate (TER) or any grading scale assessing OIC-related symptom improvement.
5. Study design (S): Randomized controlled trials.

Studies were excluded if they met any of the following criteria: 1) non-clinical studies (e.g., animal studies or *in vitro* studies), case series, conference abstracts, or theses; 2) inability to obtain valid data or access to full-text; 3) studies using interventions for the prevention rather than treatment of OIC; 4) studies using a combination of THM and CM as the experimental intervention; and 5) studies using intravenous, external applications (e.g., glycerin enema), or THM as the control intervention.

### 2.3 Outcome measures

The primary outcome was TER, which was defined as the proportion of participants who were classified as “cured”, “significantly improved”, or “improved” divided by the total number of randomized participants. These categories were generally defined by a combination of normalized or clearly improved stool frequency, shorter defecation intervals (typically within 48–72 h), softer stool form, and relief of accompanying symptoms, whereas “invalid” category indicated little or no change in bowel habits or related symptoms. Thus, TER represents the percentage of patients who showed any response other than “invalid”. TER was adopted as the primary outcome because it is widely used to reflect treatment effectiveness in the randomized trials included in this review.

Secondary outcomes were stool form, difficulty of defecation, and defecation time. Symptom severity was evaluated on a 0–4 scale. Stool form was scored as 0 for normal stool, two for dry stool, and four for pellet-like stool. Difficulty of defecation was scored as 0 for easy passage, two for straining, but self-manageable passage, and four for requiring manual assistance. Defecation time was scored as 0 for less than 10 min, two for 10–20 min, and four for >20 min (National Administration of Traditional Chinese Medicine, 2002). Additional secondary outcomes included quality of life, as measured by the Karnofsky Performance Scale (KPS) and any adverse events reported during the treatment phase.

### 2.4 Data extraction

Two investigators independently extracted data from the selected studies. The following information was extracted: first author, year of publication, sample size, cancer type, OIC diagnostic criteria, intervention (composition and duration), comparison, outcome measurements, and adverse events. Any discrepancies were resolved through discussion and consensus or, if necessary, adjudication by a third reviewer. All herbs were taxonomically validated using the Medicinal Plant Names Services (MPNS) and Plants of the World Online (POWO).

### 2.5 Quality assessment

Two reviewers independently assessed the risk of bias in the included RCTs using the Cochrane risk of bias tool. Each study was evaluated as having a low, unclear, or high risk of bias for the following domains: allocation sequence, allocation concealment, blinding of participants and personnel, blinding of outcome assessment, incomplete outcome data, selective reporting, and other bias (Higgins et al., 2024). In cases of disagreement between the two reviewers, a third reviewer made the final assessment and reached a conclusion.

### 2.6 Statistical analysis

Data analysis was conducted using Review Manager (RevMan, Version 5.4.1, Copenhagen: The Nordic Cochrane Centre, The Cochrane Collaboration, 2014). For dichotomous variables, the effect size was calculated using the risk ratio (RR) with a 95% confidence interval (CI), whereas for continuous variables, the mean difference (MD) with a 95% CI was used. Heterogeneity among studies was assessed using the Cochrane Chi-square test with a significance level of 0.10, and the I^2^ statistic, with I^2^ values >50% indicating substantial heterogeneity (Higgins et al., 2003). If more than four studies were included in each comparison and significant statistical heterogeneity (I^2^ > 50%) was observed, a random-effects model was applied; otherwise, a fixed-effects model was used (Tufanaru et al., 2015). A subgroup analysis was conducted to evaluate the validity of the studies when heterogeneity was observed. A funnel plot was constructed to assess the potential publication bias if more than 10 studies were included. In cases where asymmetry was observed, the Egger’s regression test was conducted using R version 4.4.3 to assess the presence of small study effects (Lin and Chu, 2018). In addition, the trim-and-fill method was used to adjust for publication bias when asymmetry was detected in the funnel plot, and the Egger’s test indicated potential bias. The certainty of evidence was assessed using the Grading of Recommendations Assessment, Development, and Evaluation (GRADE) approach. Each outcome was rated as “high,” “moderate,” “low,” or “very low” quality based on considerations of risk of bias, inconsistency, indirectness, imprecision, and publication bias (Atkins et al., 2004).

## 3 Results

### 3.1 Study selection

**FIGURE 1:**
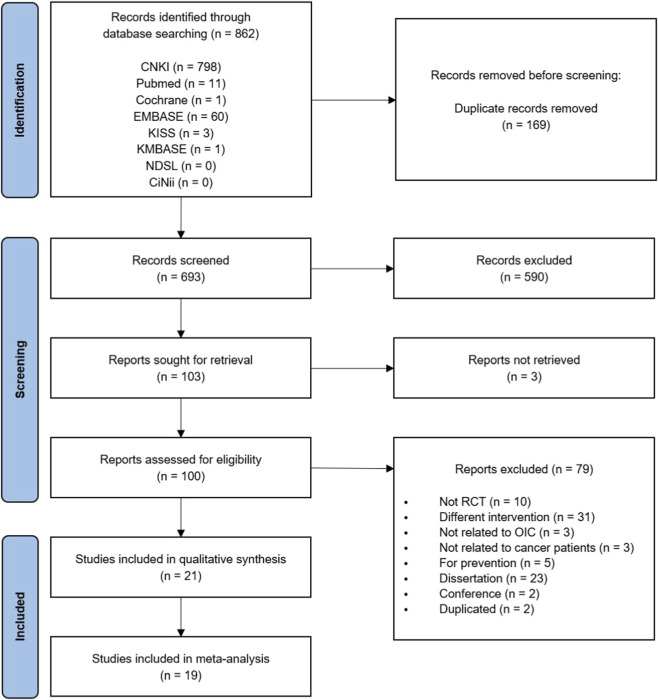
PRISMA flowchart of study selection. RCT, randomized controlled trials; OIC, opioid-induced constipation.

The initial search identified 862 studies, of which 169 were duplicates. After screening titles and abstracts, 590 studies were excluded; three studies were excluded because the full text was unavailable. The remaining 100 studies were assessed for eligibility, of which 79 were excluded for the following reasons: not RCTs (n = 10), different interventions (n = 31), not related to OIC (n = 3), not related to cancer patients (n = 3), prevention-focused rather than therapeutic use (n = 5), dissertations (n = 23), conference studies (n = 2), and duplicated publications (n = 2). Finally, 21 studies were included in the systematic review (Long and Mo, 2006; Jia et al., 2009; Gao et al., 2010; Li et al., 2012; Li, 2013; Chen et al., 2014; Sun et al., 2015; Chen, 2016; Ma et al., 2016; Zhang et al., 2016; Hou et al., 2017; Li et al., 2017; Wang, 2017; Zhu, 2017; Wu and Li, 2018; Yi et al., 2018; Chen et al., 2019; Peng et al., 2019; Yin et al., 2019; Wei and Du, 2020; Liu et al., 2022), of which 19 studies were included in the meta-analysis (Long and Mo, 2006; Jia et al., 2009; Gao et al., 2010; Li et al., 2012; Li, 2013; Chen et al., 2014; Sun et al., 2015; Chen, 2016; Zhang et al., 2016; Hou et al., 2017; Li et al., 2017; Wang, 2017; Zhu, 2017; Wu and Li, 2018; Yi et al., 2018; Peng et al., 2019; Yin et al., 2019; Wei and Du, 2020; Liu et al., 2022). A detailed flow-chart of the study selection process is shown in Figure 1.

### 3.2 Study characteristics

Characteristics of the included RCTs are summarized in Table 1. All studies were published between 2006 and 2022. The sample size ranged from 46 to 406, with 2,108 patients. Patients with various cancer types (e.g., lung, breast, pancreatic cancer) participated in 15 studies, while six studies did not specify the cancer type. One study enrolled patient with lung cancer (Ma et al., 2016). Six studies excluded patients with gastrointestinal cancer because of its potential impact on intestinal function and stool form (Table 1). Two studies included patients with stage III or IV (n = 2) (Long and Mo, 2006; Wang, 2017), one study included patients with stage II, III, or IV (n = 1) (Ma et al., 2016), and one study included patients with stage I, II, or III (n = 1) (Sun et al., 2015). Nine studies did not specify types of opioid analgesics (Jia et al., 2009; Gao et al., 2010; Li, 2013; Sun et al., 2015; Hou et al., 2017; Wang, 2017; Chen et al., 2019; Peng et al., 2019; Liu et al., 2022); the remaining studies addressed the specific opioid analgesics. Morphine was used in six studies (Long and Mo, 2006; Li et al., 2012; Ma et al., 2016; Zhu, 2017; Yi et al., 2018; Wei and Du, 2020), oxycodone in three (Chen et al., 2014; Chen, 2016; Yin et al., 2019), hydrocodone in one study (Wu and Li, 2018), meperidine in one study (Zhang et al., 2016), and oxycodone or methadone in one study (Li et al., 2017).

**TABLE 1:** Basic characteristics of included studies.

Study id	Sample size (I/C)	Cancer type	Criteria	Intervention	Control	Duration (days)	Outcome	Statistical results	Adverse events
Chen (2016)	54/54	Various	ROME III	Zengye Chengqi Dec.	Lactulose	7	TER	TER RR = 1.16 [1.00, 1.34]	N/A
Gao et al. (2010)	42/20	Various	N/A	Yiqi Runchang Dec.	Lactulose	28	TER, CS, BM, AS	TER RR = 1.04 [0.81, 1.35]	N/A
Hou et al. (2017)	24/24	N/A	N/A	Maziren Dec.	Lactulose	14	TER, FDT, DI, DT, SF, DD, NRS, KPS	TER RR = 1.38 [1.01, 1.87] SF MD = −0.39 [-0.62, −0.16] DD MD = −0.41 [-0.75, −0.07] DT MD = −0.45 [-0.75, −0.15] KPS MD = 4.89 [1.92, 7.86]	N/A
Li et al. (2012)	33/33	Various	N/A	Maziren Pill	Lactulose	14	FDT DI, DT, SF DD, ER, KPS, NRS	TER RR = 1.07 [0.88, 1.32] SF MD = 0.36 [0.22, 0.50] DD MD = −0.18 [-0.37, 0.01] DT MD = −0.29 [-0.69, −0.11]	N/A
Li et al. (2017)	44/44	Various (GI cancer excluded)	>3days with abdominal symptoms	Zhe Sui Gongjie Tang	Lactulose	14	TER, VAS, KPS, GIQLI	TER RR = 1.24 [1.04, 1.47] KPS MD = 9.00 [7.21, 10.79]	N/A
Liu et al. (2022)	30/30	Various (GI cancer excluded)	ROME IV	Sini San with Suzi Jiangqi Tang	Lactulose	28	TER, DF, DD. DT. SF, AS, PAC-QOL	TER RR = 1.17 [0.93, 1.48] SF MD = 0.10 [-0.32, 0.52] DD MD = −0.40 [-0.75, −0.05] DT MD = −0.30 [-0.67, 0.07]	N/A
Peng et al. (2019)	50/50	N/A	ROME III	Jiawei Sini Dec.	Lactulose	21	DF, BSS, CS, FDT, DI, DT, SF, DD, NRS, PAC-QOL	SF MD = −0.41 [-0.50, −0.32] DD MD = −0.51 [-0.62, −0.40] DT MD = −0.47 [-0.59, −0.35]	N/A
Wei and Du (2020)	30/30	Various	domestic guidelines	Yiqi Zengye Dec.	Lactulose	14	TER, AS, KPS	TER RR = 1.09 [0.84, 1.40]	N/A
Wu and Li (2018)	30/30	Various	ROME III	Xiaoji Anzhong Dec.	Lactulose	14	TER, AS, KPS	TER RR = 1.23 [0.96, 1.57]	N/A
Yi et al. (2018)	30/30	Various (GI cancer excluded)	ROME III	Qirong Runchang Liq	Lactulose	7	DI, DT, SF, DD, ER, AS	TER RR = 1.22 [0.85, 1.76] SF MD = 0.00 [-0.19, 0.19] DD MD = 0.20 [-0.06, 0.46] DT MD = 0.10 [-0.23, 0.42]	N/A
Yin et al. (2019)	30/30	Various (GI cancer excluded)	domestic guidelines	Zengye Chengqi Dec.	Lactulose	7	TER, DF, DT, SF, DD, AS	TER RR = 1.26 [1.02, 1.55] SF MD = −0.50 [-0.91, −0.09] DD MD = −0.64 [-0.98, −0.30] DT MD = −0.13 [-0.50, 0.24]	N/A
Zhang et al. (2016)	33/33	Various	ROME III	Yangyin Liqi Dec.	Lactulose	14	TER, CCS, BUP, NRS, AEs, BT	TER RR = 1.30 [1.02, 1.67]	diarrhea (n = 1)
Zhu (2017)	30/30	Various	domestic guidelines	Tongxia Runchang Dec.	Lactulose	14	TER, AEs	TER RR = 1.35 [1.02, 1.79]	diarrhea (n = 2), nausea (n = 1)
Chen et al. (2014)	203/203	Various (GI cancer excluded)	ROME III	Xiaochengqi Dec. with Zengye Dec.	Phenolphthalein	14	CCS, ER, EORTC QLQ-C30, NRS, AEs	TER RR = 1.08 [1.01, 1.16]	diarrhea (n = 9), nausea (n = 2), abdominal pain (n = 1)
Li (2013)	32/30	Various	domestic guidelines	Qizhu Zengye Dec.	Phenolphthalein	14	TER	TER RR = 1.56 [1.15, 2.12]	N/A
Long and Mo (2006)	130/102	Various	symptom-based criteria	Xin Jia Huang Long Tang	Phenolphthalein	N/A	TER	TER RR = 1.42 [1.21, 1.67]	N/A
Jia et al. (2009)	43/35	N/A	>3days with abdominal symptoms	Jiangni Kuanchang Dec.	Aloe Capsule	28	TER, DI, SF, DD, AS, KPS, BW, BT	TER RR = 1.33 [1.07, 1.66] SF MD = −0.09 [-0.31, 0.13] DD MD = −0.39 [-0.65, −0.13]	No AEs
Sun et al. (2015)	23/23	Multiple Various (GI cancer excluded)	>3days with abdominal symptoms	Liqi Jiangni Gran	Aloe capsule	7	KPS, DF, DT, SF, DD, ER, AS	TER RR = 1.21 [0.86, 1.71] SF MD = −0.44 [-0.77, −0.11] DD MD = −0.18 [-0.47, 0.11] DT MD = −0.18 [-0.69, 0.33]	N/A
Ma et al. (2016)	105/106	Lung cancer	N/A	Huisheng Sol	Mosapride citrate	N/A	AD	N/A	N/A
Wang (2017)	23/23	Various	domestic guidelines	Fuzheng Runchang Dec.	Mosapride citrate	20	TER, KPS, AEs	TER RR = 1.17 [0.91, 1.50] KPS MD = 5.93 [4.21, 7.65]	No AEs
Chen et al. (2019)	65/64	Various	International consensus	Bianmitong	Placebo	14	SBM, AEs, COWS, NRS	N/A	dyspepsia, abdominal discomfort

AD, amount of defecation; AEs, adverse events; AS, accompanying symptoms; BM, time to first bowel movement; BSS, bristol stool scale; BT, blood test; BUP, brain gut peptide; BW, body weight; C, control; CCS, cleveland constipation score; CI, confidence Interval; COWS, clinical opiate withdrawal scale; CS, constipation score; DD, difficulty of in defecation; Dec, Decoction; DF, defecation frequency; DI, defecation interval; DT, defecation time; EORTC QLQ-C30, European Organisation for Research and Treatment of Cancer Quality of Life Questionnaire-Core 30; FDT, first defecation time; Gastrointestinal Quality of Life Index, GIQLI; gran, Granule; I, intervention; KPS, karnofsky performance score; Liq, Liquid; MD, mean difference; NRS, numeric rating scale of pain score; PAC-QOL, patient assessment of constipation quality of life; RR, risk ratio; SBM, spontaneously bowel movement rate; SF, stool forms; Sol, Solution; TER, total effective rate; VAS, visual analog scale of pain score.

The diagnostic criteria for constipation varied across studies. Seven studies used the ROME criteria (III or IV), and five studies used Chinese domestic guidelines. Three studies defined constipation as >3 days with abdominal symptoms, one study relied on symptom-based criteria without specifying the duration, and one study applied a criterion based on the international consensus statement. Four studies did not report the specific diagnostic criteria (Table 1).

Eight studies included patients with pattern identifications, with the following subgroup classifications: Qi-stagnation (n = 3) (Sun et al., 2015; Zhang et al., 2016; Liu et al., 2022); Yin-deficiency (n = 3) (Zhang et al., 2016; Wu and Li, 2018; Yin et al., 2019); Stomach-heat (n = 2) (Li et al., 2012; Sun et al., 2015); Qi-Yin deficiency (n = 1) (Gao et al., 2010); Spleen-Stomach Qi deficiency (n = 1) (Li et al., 2012); and Spleen-Kidney Yang deficiency (n = 1) (Peng et al., 2019). Three studies included patients with multiple pattern identifications, such as Stomach-dryness and heat and Spleen-Stomach Qi deficiency (Li et al., 2012; Sun et al., 2015; Zhang et al., 2016). Other studies did not specify any pattern identifications.

THM prescriptions vary across studies in terms of formula and composition, indicating substantial heterogeneity. Detailed information is provided in Supplementary Material S2. The most frequently used herbs were *Citrus × aurantium f. aurantium* [Rutaceae; Aurantii Immaturus Fructus], which appeared in 15 prescriptions. *Rehmannia glutinosa (Gaertn.) Libosch. ex DC.* [Orobanchaceae; Rehmanniae Radix] and *Angelica gigas Nakai* [Apiaceae; Angelicae Gigantis Radix] were each used in 11 prescriptions. Herbs such as *Rheum officinale Baill.* [Polygonaceae; Rhei Rhizoma], *Atractylodes lancea (Thunb.) DC.* [Asteraceae; Atractylodis Rhizoma Alba], *Scrophularia ningpoensis Hemsl.* [Scrophulariaceae; Scrophulariae Radix] and *Cannabis sativa L.* [Cannabaceae; Cannabis Semen] appeared in nine prescriptions. The main mechanisms of these frequently used herbs and the prescriptions in which they were included are summarized in Table 2.

**TABLE 2:** Frequency analysis of herbs in the included prescriptions.

Scientific name	Frequency of herbs	Main actions	References
*Citrus × aurantium f. aurantium* [Rutaceae; Aurantii Immaturus Fructus]	15	↑intestinal transit; Ca^2+^-dependent smooth muscle contraction/ICCs modulation (Tan et al., 2017; Wen et al., 2024). potential mild induction of CYP1A2 and CYP3A4; p-synephrine appear to have low risk of clinically significant drug interactions (Zhou et al., 2018; Stohs et al., 2012)	Zengye Chengqi Dec. (Chen, 2016), Yiqi Runchang Dec. (Gao et al., 2010), Maziren Dec. (Hou et al., 2017), Maziren Pill (Li et al., 2012), Sini San with Suzi Jiangqi Tang (Liu et al., 2022), Jiawei Sini Dec. (Peng et al., 2019), Xiaoji Anzhong Dec. (Wu and Li, 2018), Qirong Runchang Liq. (Yi et al., 2018), Tongxia Runchang Dec. (Zhu, 2017), Xiaochengqi Dec. with Zengye Dec. (Chen et al., 2014), Qizhu Zengye Dec. (Li, 2013), Jiangni Kuanchang Dec. (Jia et al., 2009), Liqi Jiangni Gran. (Sun et al., 2015), Fuzheng Runchang Dec. (Wang, 2017), Bianmitong (Chen et al., 2019)
*Rehmannia glutinosa (Gaertn.) Libosch. ex DC.* [Orobanchaceae; Rehmanniae Radix]	11	protects intestinal mucosal barrier; modulates gut microbiota and short-chain fatty acids (Lv et al., 2023; Li et al., 2023)	Zengye Chengqi Dec. (Chen, 2016), Yiqi Runchang Dec. (Gao et al., 2010), Yiqi Zengye Dec. (Wei and Du, 2020), Xiaoji Anzhong Dec. (Wu and Li, 2018), Qirong Runchang Liq. (Yi et al., 2018), Zengye Chengqi Dec. (Yin et al., 2019), Yangyin Liqi Dec. (Zhang et al., 2016), Xiaochengqi Dec. with Zengye Dec. (Chen et al., 2014), Qizhu Zengye Dec. (Li, 2013), Xin Jia Huang Long Dec. (Long and Mo, 2006), Fuzheng Runchang Dec. (Wang, 2017)
*Angelica gigas Nakai* [Apiaceae; Angelicae Gigantis Radix]	11	↑GI motility (ICCs, motilin); anti-inflammatory/anti-oxidant (Cho et al., 2015; Kwon et al., 2021; Batiha et al., 2022).Warfarin-induced anticoagulation via coumarin-like components and antiplatelet activity (Zhuang et al., 2021)	Zengye Chengqi Dec. (Chen, 2016), Maziren Dec. (Hou et al., 2017), Maziren Pill (Li et al., 2012), Sini San with Suzi Jiangqi Tang (Liu et al., 2022), Jiawei Sini Dec. (Peng et al., 2019), Yiqi Zengye Dec. (Wei and Du, 2020), Qirong Runchang Liq. (Yi et al., 2018), Tongxia Runchang Dec. (Zhu, 2017), Qizhu Zengye Dec. (Li, 2013), Xin Jia Huang Long Dec. (Long and Mo, 2006), Fuzheng Runchang Dec, (Wang, 2017)
*Rheum officinale Baill.* [Polygonaceae; Rhei Rhizoma]	9	stimulant laxative (anthraquinones; ↑motility); ↑intestinal fluid secretion (Gordon et al., 2016; Lv et al., 2024); risk of hypokalemia with long-term or high-dose use, potentially enhanced by concomitant potassium-depleting medications (e.g., diuretics) (EMA, 2022)	Maziren Dec. (Hou et al., 2017), Maziren Pill (Li et al., 2012), Zengye Chengqi Dec. (Yin et al., 2019), Yangyin Liqi Dec. (Zhang et al., 2016), Tongxia Runchang Dec. (Zhu, 2017), Xiaochengqi Dec. with Zengye Dec. (Chen et al., 2014), Xin Jia Huang Long Dec. (Long and Mo, 2006), Jiangni Kuanchang Dec. (Jia et al., 2009), Bianmitong (Chen et al., 2019)
*Atractylodes lancea (Thunb.) DC.* [Asteraceae; Atractylodis Rhizoma Alba]	9	modulates colonic motility (ICCs, smooth muscle); gut microbiota-SCFAs-5-HT axis (Sung et al., 2025; Gao et al., 2025)	Zengye Chengqi Dec. (Chen, 2016), Sini San with Suzi Jiangqi Tang (Liu et al., 2022), Jiawei Sini Dec. (Peng et al., 2019), Yiqi Zengye Dec. (Wei and Du, 2020), Qirong Runchang Liq. (Yi et al., 2018), Tongxia Runchang Dec. (Zhu, 2017), Qizhu Zengye Dec. (Li, 2013), Fuzheng Runchang Dec, (Wang, 2017), Bianmitong (Chen et al., 2019)
*Scrophularia ningpoensis Hemsl.* [Scrophulariaceae; Scrophulariae Radix]	9	↑intestinal fluid secretion; modulates gut motility via SCF/c-Kit-ICC pathway (Yao et al., 2025; Lv et al., 2025)	Zengye Chengqi Dec. (Chen, 2016), Yiqi Runchang Dec. (Gao et al., 2010), Yiqi Zengye Dec. (Wei and Du, 2020), Qirong Runchang Liq. (Yi et al., 2018), Zengye Chengqi Dec. (Yin et al., 2019), Yangyin Liqi Dec. (Zhang et al., 2016), Xiaochengqi Dec. with Zengye Dec. (Chen et al., 2014), Qizhu Zengye Dec. (Li, 2013), Xin Jia Huang Long Dec. (Long and Mo, 2006)
*Cannabis sativa L.* [Cannabaceae; Cannabis Semen]	9	↑intestinal motility and water-electrolyte balance; modulates gut microbiota (Li et al., 2022) potential CYP450-related interactions, particularly with antiepileptics, warfarin, and tacrolimus (Ho et al., 2024)	Zengye Chengqi Dec. (Chen, 2016), Yiqi Runchang Dec. (Gao et al., 2010), Maziren Dec. (Hou et al., 2017), Maziren Pill (Li et al., 2012), Yiqi Zengye Dec. (Wei and Du, 2020), Xiaoji Anzhong Dec. (Wu and Li, 2018), Qirong Runchang Liq. (Yi et al., 2018), Yangyin Liqi Dec. (Zhang et al., 2016), Tongxia Runchang Dec. (Zhu, 2017)
*Ophiopogon japonicus (Thunb.) Ker Gawl.* [Asparagaceae; Liriopis Tuber]	8	↑mucus/secretions, protects mucosa (Kim et al., 2016)	Zengye Chengqi Dec. (Chen, 2016), Yiqi Zengye Dec. (Wei and Du, 2020), Qirong Runchang Liq. (Yi et al., 2018), Zengye Chengqi Dec. (Yin et al., 2019), Yangyin Liqi Dec. (Zhang et al., 2016), Xiaochengqi Dec. with Zengye Dec. (Chen et al., 2014), Qizhu Zengye Dec. (Li, 2013), Xin Jia Huang Long Dec. (Long and Mo, 2006)
*Magnolia officinalis Rehder and E.H.Wilson* [Magnoliaceae; Magnoliae Cortex]	8	prokinetic/antispasmodic; ↓bloating, gas (Zhang et al., 2005; Wang et al., 2019)	Maziren Dec. (Hou et al., 2017), Maziren Pill (Li et al., 2012), Sini San with Suzi Jiangqi Tang (Liu et al., 2022), Jiawei Sini Dec. (Peng et al., 2019), Tongxia Runchang Dec. (Zhu, 2017), Xiaochengqi Dec. with Zengye Dec. (Chen et al., 2014), Jiangni Kuanchang Dec. (Jia et al., 2009), Liqi Jiangni Gran. (Sun et al., 2015)
*Astragalus mongholicus Bunge* [Fabaceae; Astragali Radix]	7	gut barrier/immune modulation (Su et al., 2023)	Maziren Dec. (Hou et al., 2017), Maziren Pill (Li et al., 2012), Jiawei Sini Dec. (Peng et al., 2019), Yiqi Zengye Dec. (Wei and Du, 2020), Xiaoji Anzhong Dec. (Wu and Li, 2018), Qirong Runchang Liq. (Yi et al., 2018), Qizhu Zengye Dec. (Li, 2013)
*Paeonia lactiflora Pall.* [Paeoniaceae; Paeoniae Radix Alba]	6	improves defecation frequency, and soften the stool; reduce colonic inflammation (↓ NO/NOS, IL-1β, TNF-α, NF-kB, SP), enhance motility (Liu et al., 2020)	Zengye Chengqi Dec. (Chen, 2016), Maziren Dec. (Hou et al., 2017), Maziren Pill (Li et al., 2012), Sini San with Suzi Jiangqi Tang (Liu et al., 2022), Qizhu Zengye Dec. (Li, 2013), Fuzheng Runchang Dec, (Wang, 2017)
*Prunus armeniaca L.* [Rosaceae; Armeniacae Semen]	6	oil-containing stool softener; mild prokinetic effect (Mahboubi, 2021)	Yiqi Runchang Dec. (Gao et al., 2010), Maziren Pill (Li et al., 2012), Sini San with Suzi Jiangqi Tang (Liu et al., 2022), Xiaoji Anzhong Dec. (Wu and Li, 2018), Tongxia Runchang Dec. (Zhu, 2017), Fuzheng Runchang Dec, (Wang, 2017)

5-HT, 5-hydroxytryptamine (serotonin); c-Kit, tyrosine-protein kinase Kit (CD117); CYP, cytochrome P450; Dec, decoction; GI, gastrointestinal; Gran, granule; Sol, solution; ICCs, interstitial cells of Cajal; IL-1β, interleukin-1 β; Liq, liquid; NF-kB, nuclear factor-kB; NO, nitric oxide; NOS, nitric oxide synthase; SCF, stem cell factor; SCFAs, short-chain fatty acids; SP, substance P; TNF- α, tumor necrosis factor-α.

The control groups were administered CM such as lactulose, phenolphthalein, aloe capsules, or mosapride citrate, and one trial used placebo as the comparator (Table 1). Most studies assessed the effectiveness of THM using TER, while several studies assessed stool form and difficulty of defecation, defecation time or interval, and defecation frequency (Table 1). Quality of life was commonly measured with KPS, and less frequently with instruments such as the Patients Assessment of Constipation Quality of Life (PAC-QOL), Gastrointestinal Quality of Life Index (GIQLI), and European Organisation for Research and Treatment of Cancer Quality of Life Questionnaire-Core 30 (EORTC QLQ-C30), together with other outcomes including Cleveland Constipation Score (CCS), Clinical Opiate Withdrawal Scale (COWS), Numeric Rating Scale of Pain Score (NRS), Visual Analog Scale of Pain Score (VAS), Bristol Stool Scale (BSS), and accompanying symptoms such as spontaneous bowel movement, nausea, and abdominal pain (Table 1).

### 3.3 Risk of bias in the included studies

**FIGURE 2:**
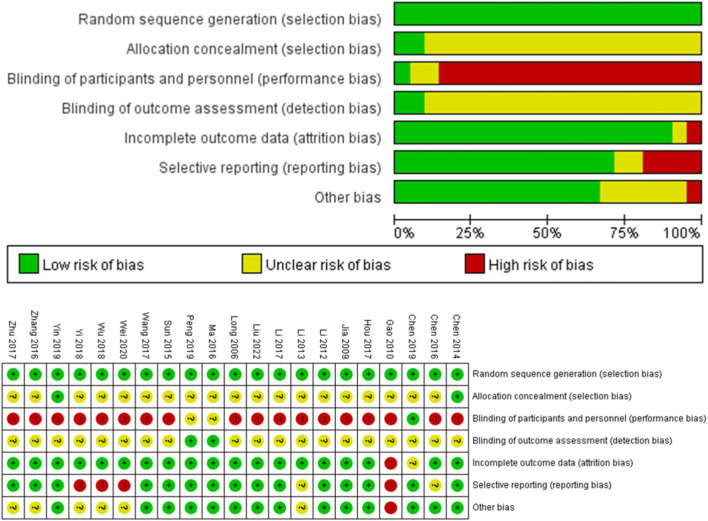
Risk of Bias in the included studies graph (upper) and summary (lower). +, low risk of bias; ?, unclear of bias; -, high risk of bias.

The risk of bias in the included studies is presented in Figure 2. All studies adequately described random sequence generation. In terms of allocation concealment, two studies reported the detailed procedures, while the remaining 19 studies had an uncertain bias. Eighteen studies did not blind the participants and personnel, while two studies were assessed as unclear. However, one study using a placebo as the control was assessed as having a low risk of performance bias (Figure 2).

Two studies were evaluated as low risk for blinding of outcomes assessment, whereas 19 studies were rated as unclear. Nineteen studies had a low risk of incomplete outcome data, whereas one study had an unclear risk. One study was assessed as high risk owing to discrepancies between the number of participants initially recruited and those included in the final analysis without explanation. Regarding reporting bias, two studies were unclear, four studies were high risk, and the remaining 15 studies were low risk. Except for seven studies, 14 studies were considered to have a low risk of other biases because of no significant baseline differences between the intervention and control groups (Figure 2).

### 3.4 THM versus CM

#### 3.4.1 TER

**FIGURE 3:**
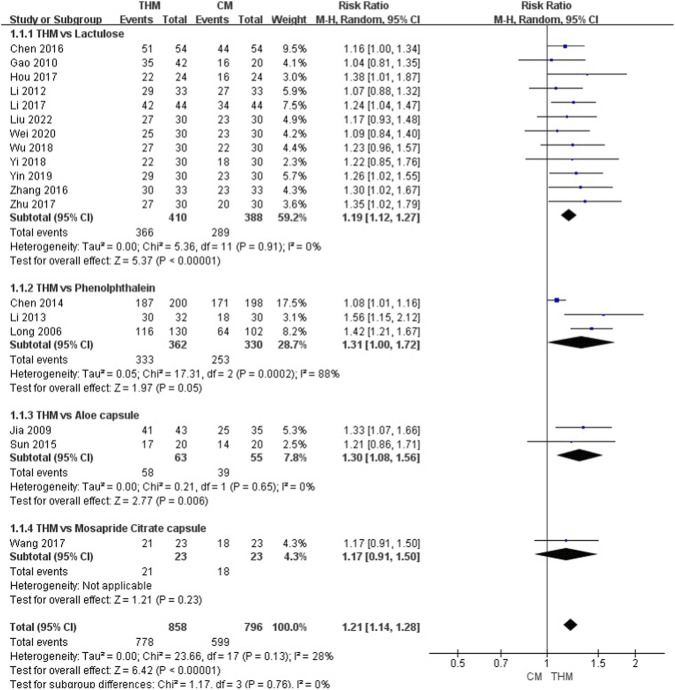
Forest plot of the comparison of THM versus CM for total effective rate. THM, traditional herbal medicine; CM, conventional medicine.

Eighteen RCTs involving 1,654 patients were included in the TER meta-analysis (Figure 3) (Long and Mo, 2006; Jia et al., 2009; Gao et al., 2010; Li et al., 2012; Li, 2013; Chen et al., 2014; Sun et al., 2015; Chen, 2016; Zhang et al., 2016; Hou et al., 2017; Li et al., 2017; Wang, 2017; Zhu, 2017; Wu and Li, 2018; Yi et al., 2018; Yin et al., 2019; Wei and Du, 2020; Liu et al., 2022). Overall, THM demonstrated a statistically significant improvement compared to CM (RR 1.21, 95% CI 1.14–1.28, p < 0.01), with low heterogeneity among studies (I^2^ = 28%).

In the subgroup analysis, THM showed a significant improvement compared to lactulose (RR 1.19, 95% CI 1.12–1.27, p < 0.01), with low heterogeneity (I^2^ = 0%) (Gao et al., 2010; Li et al., 2012; Chen, 2016; Zhang et al., 2016; Hou et al., 2017; Li et al., 2017; Zhu, 2017; Wu and Li, 2018; Yi et al., 2018; Yin et al., 2019; Wei and Du, 2020; Liu et al., 2022). Furthermore, THM showed significant improvement compared to phenolphthalein (RR 1.31, 95% CI 1.00–1.72, p = 0.05), with high heterogeneity among studies (I^2^ = 88%) (Long and Mo, 2006; Li, 2013; Chen et al., 2014) and showed significantly improved symptoms compared to aloe capsules (RR = 1.30, 95% CI 1.08–1.56, p < 0.01), with low grade of heterogeneity (I^2^ = 0%) (Jia et al., 2009; Sun et al., 2015). However, THM did not show a statistically significant improvement compared to mosapride citrate (RR 1.17, 95% CI 0.91–1.50, p = 0.23); because only one study was included in the analysis, heterogeneity could not be assessed (Wang, 2017). According to the GRADE assessment, the certainty of evidence for TER was rated high (Table 3).

**TABLE 3:** Summary of findings.

THM compared to CM for OIC in patients with cancer									
Patient or population: Patients with OIC in cancer Intervention: THM Comparison: CM									
Outcomes	Anticipated absolute effects^a^ (95% CI)		Certainty assessment					No. of participants (studies)	Certainty of the evidence (GRADE)
	Risk with CM	Risk with THM	Risk of bias	Inconsistency	Indirections	Imprecision	Other consideration		
TER	753 per 1,000	911 per 1,000 (858–963)	Not serious	Not serious	Not serious	Not serious	None	1,654 (18 RCTs)	⊕⊕⊕⊕ High
SF	The mean stool form was 0	MD 0.16 lower (0.43 lower to 0.1 higher)	Not serious	Very serious	Not serious	Serious	None	512 (8 RCTs)	⊕○○○ Very low
DD	The mean difficulty of defecation was 0	MD 0.31 lower (0.49 lower to 0.13 lower)	Not serious	Very serious	Not serious	Not serious	None	518 (8 RCTs)	⊕⊕○○ Low
DT	The mean defecation time was 0	MD 0.28 lower (0.45 lower to 0.1 lower)	Not serious	Serious	Not serious	Not serious	None	434 (7 RCTs)	⊕⊕⊕○ Moderate
KPS	The mean Karnofsky Performance Score was 0	MD 6.76 higher (4.32 higher to 9.2 higher)	Not serious	Very serious	Not serious	Not serious	None	182 (3 RCTs)	⊕⊕○○ Low

^a^ The risk in the intervention group (and its 95% confidence interval) is based on the assumed risk in the comparison group and the relative effect of the intervention (and its 95% CI).

CI, confidence interval; CM, conventional medicine; DD, difficulty of defecation; DT, defecation time; KPS, karnofsky performance scale; MD, mean difference; RR, risk ratio; SF, stool form; TER, total effective rate; THM, traditional herbal medicine.

#### 3.4.2 Stool form

**FIGURE 4:**
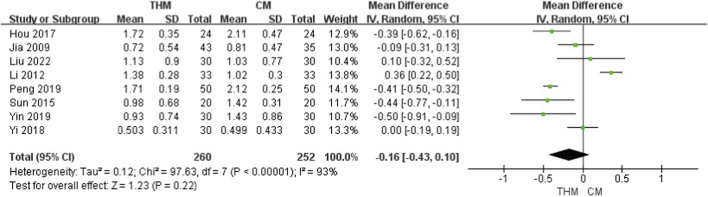
Forest plot of the comparison of THM versus CM for stool form. THM, traditional herbal medicine; CM, conventional medicine.

Eight RCTs with a total of 512 patients assessed the effect of THM versus CM on stool form and were included in the meta-analysis (Figure 4) (Jia et al., 2009; Li et al., 2012; Sun et al., 2015; Hou et al., 2017; Yi et al., 2018; Peng et al., 2019; Yin et al., 2019; Liu et al., 2022). THM did not show a significant improvement in stool form compared to CM (MD –0.16, 95% CI – 0.43–0.10, p = 0.22), with high heterogeneity among studies (I^2^ = 93%). According to the GRADE assessment, the certainty of evidence for this outcome was rated as very low because of inconsistency and imprecision (Table 3).

#### 3.4.3 Difficulty of defecation

**FIGURE 5:**
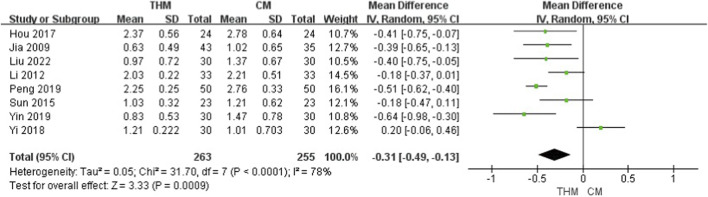
Forest plot of the comparison of THM versus CM for difficulty of defecation. THM, traditional herbal medicine; CM, conventional medicine.

Eight RCTs with a total of 518 patients were included to evaluate the effect of THM versus CM on defecation difficulty (Figure 5) (Jia et al., 2009; Li et al., 2012; Sun et al., 2015; Hou et al., 2017; Yi et al., 2018; Peng et al., 2019; Yin et al., 2019; Liu et al., 2022). THM showed a statistically significant improvement compared to CM (MD –0.31, 95% CI –0.49 to −0.13, p < 0.01), with a high heterogeneity (I^2^ = 78%). The GRADE assessment indicated that the certainty of the evidence was low because of inconsistencies among studies (Table 3).

#### 3.4.4 Defecation time

**FIGURE 6:**
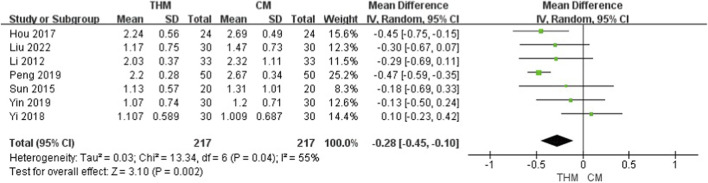
Forest plot of the comparison of THM versus CM for defecation time. THM, traditional herbal medicine; CM, conventional medicine.

Seven RCTs with a total of 434 patients reported effects of THM versus CM for every defecation time (Figure 6) (Li et al., 2012; Sun et al., 2015; Hou et al., 2017; Yi et al., 2018; Peng et al., 2019; Yin et al., 2019; Liu et al., 2022). THM significantly reduced defecation time compared to CM (MD –0.28, 95% CI –0.45 to −0.10, p < 0.01), with moderate heterogeneity (I^2^ = 55%). The GRADE assessment indicated that the certainty of evidence was moderate owing to inconsistencies among studies (Table 3).

#### 3.4.5 KPS

**FIGURE 7:**
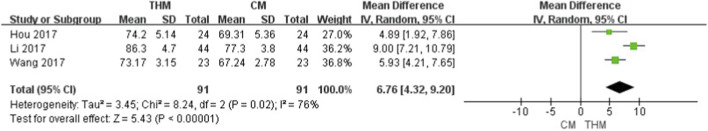
Forest plot of the comparison of THM versus CM for Karnofsky Performance Scale scores. THM, traditional herbal medicine; CM, conventional medicine.

Three RCTs with a total of 182 patients were included in the meta-analysis comparing THM and CM using KPS (Figure 7) (Jia et al., 2009; Li et al., 2012; Sun et al., 2015; Hou et al., 2017; Li et al., 2017; Wang, 2017; Wei and Du, 2020). THM showed a statistically significant improvement (MD 6.76, 95% CI 4.32–9.20, p < 0.01), with high heterogeneity (I^2^ = 76%) (Hou et al., 2017; Li et al., 2017; Wang, 2017). The certainty of the evidence was rated low according to the GRADE assessment (Table 3).

### 3.5 THM versus placebo

Only one study compared THM with a placebo (Chen et al., 2019). In this study, the proportion of patients with improved spontaneous bowel movements (SBM) was significantly higher in the THM group (69.2%) than in the placebo group (30.3%) (p < 0.05). The THM group also demonstrated significantly greater improvements in weekly SBM frequency, complete spontaneous bowel movements (CSBM), and SBM without discomfort.

### 3.6 Adverse events

Only six RCTs reported adverse events. No severe adverse events, including grade 3 or higher on the Common Terminology Criteria for Adverse Events (CTCAE) scale, were observed. Two studies confirmed no abnormalities in liver or kidney function parameters and no significant differences between groups (Jia et al., 2009; Zhang et al., 2016). Four studies reported mild gastrointestinal symptoms, including diarrhea, nausea, and abdominal pain (Chen et al., 2014; Zhang et al., 2016; Zhu, 2017; Chen et al., 2019). In two studies, mild diarrhea was alleviated by reducing the dosage or temporarily discontinuing the treatment (Chen et al., 2014; Zhang et al., 2016). Another study reported that diarrhea and nausea improved with symptomatic treatment (Zhu, 2017). One study reported no significant adverse events in either group and found that the treatment group experienced an improved quality of life compared to the control group (Wang, 2017) (Table 1).

### 3.7 Publication bias

**FIGURE 8:**
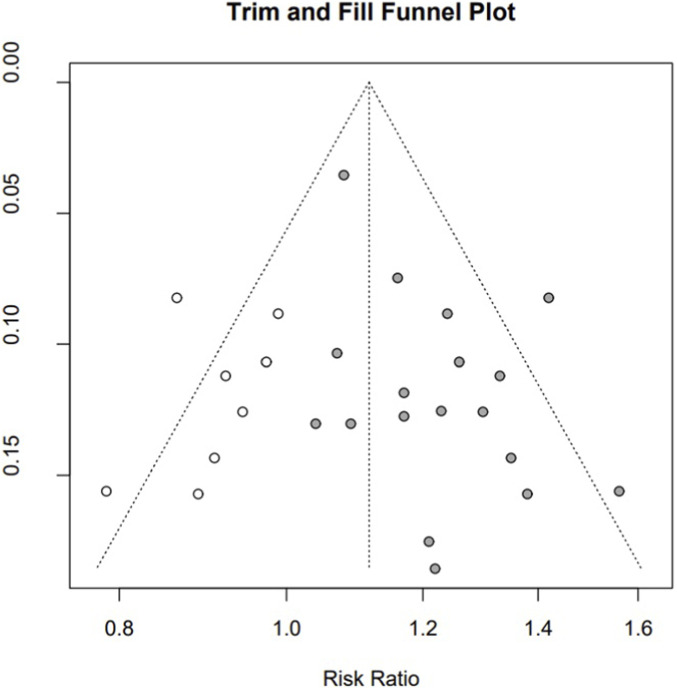
Trim-and-fill adjusted plot with the imputed studies included.

A funnel plot of 18 RCTs comparing THM with CM for TER was constructed to assess potential publication bias (Long and Mo, 2006; Jia et al., 2009; Gao et al., 2010; Li et al., 2012; Li, 2013; Chen et al., 2014; Sun et al., 2015; Chen, 2016; Zhang et al., 2016; Hou et al., 2017; Li et al., 2017; Wang, 2017; Zhu, 2017; Wu and Li, 2018; Yi et al., 2018; Yin et al., 2019; Wei and Du, 2020; Liu et al., 2022). Visual inspection of the plot suggested asymmetry, which was statistically supported by Egger’s regression test (t = 2.56, p = 0.02). A trim-and-fill method was used to account for bias. The adjusted pooled RR was 1.12 (95% CI 1.05–1.19), with the beneficial effect remaining statistically significant (Figure 8).

## 4 Discussion

This systematic review and meta-analysis evaluated the efficacy and safety of THM in the management of OIC in patients with cancer. In total, 21 studies involving 2,108 patients were reviewed, of which 19 were included in the quantitative analysis. Overall, THM significantly improved OIC symptoms compared with CM, with a higher TER and favorable effects on difficulty of defecation, defecation time, and KPS. However, considerable heterogeneity was observed for several outcomes, which is likely related to variations in prescriptions, cancer types and stages, and concomitant anticancer regimens that could not be fully standardized across trials. Consistent with this, the GRADE assessment rated the certainty of evidence as high for the composite TER, but “very low” for stool form due to inconsistency and imprecision and “low” for difficulty of defecation and KPS, mainly due to inconsistency, underscoring that these results should be interpreted with caution.

In many trials included, performance bias was assessed as high because complete blinding of participants and clinicians was difficult to achieve when using strongly flavored, aromatic herbal decoctions that are readily distinguishable from CM or placebos. Allocation concealment (selection bias) was frequently rated as unclear, as details on how the random sequence was implemented and shielded from investigators (e.g., use of opaque sealed envelopes or centralized randomization) were not reported. Similarly, the risk of detection bias was commonly unclear because few studies specified whether outcome assessors were blinded to group allocation or whether patients-reported symptom scales were collected by independent personnel. Therefore, beyond trial quality, it is also important to consider whether there is a plausible biological and traditional rationale for THM in this setting.

OIC is caused by the activation of μ-opioid receptors in the gastrointestinal tract, which inhibit peristalsis, delay gastric emptying, and promote fluid absorption (Liu and Wittbrodt, 2002; De Schepper et al., 2004; De Giorgio et al., 2021). These mechanisms result in reduced motility and difficulty in stool formation. From a traditional Eastern Asian medicine perspective, such manifestations have been described as Qi stagnation, characterized by reduced motility with straining and a sense of incomplete evacuation, and Yin deficiency, typically presenting as dry and compact stools (Lam et al., 2019). In this review, these two patterns were the most commonly reported patterns in the eight studies that incorporated traditional medicine diagnostic classification. Beyond these mechanistic considerations, preclinical and clinical studies on THM for constipation indicate several pathways through which THM may act. Herbal formulas have been shown to enhance gastrointestinal motility by modulating interstitial cells of Cajal (ICCs), enteric neural pathways and gut hormone secretion, thereby improving coordinated smooth muscle contraction in constipation models (Tan et al., 2017; Wen et al., 2024). In addition, several THM increase intestinal water and mucus secretion, which softens stools and facilitates colonic transit, suggesting that THM can target both motility and stool consistency in constipation (Gordon et al., 2016; Lv et al., 2024).

In this review, frequently used herbs showed convergent mechanisms that can explain the improvements in OIC. *Citrus × aurantium f. aurantium* [Rutaceae; Aurantii Immaturus Fructus] enhances intestinal transit through Ca^2+^-independent smooth muscle contraction and modulation of inerstitial cells of Cajal indicating a prokinetic effect. *Rehmannia glutinosa (Gaertn.) Libosch. ex DC.* [Orobanchaceae; Rehmanniae Radix] primarily protects the intestinal mucosal barrier and modulates gut microbiota suggesting a more tonic effect on epithelial integrity and the luminal environment. In addition, anthraquinone-containing *Rheum officinale Baill.* [Polygonaceae; Rhei Rhizoma] acts similarly to stimulant laxatives, while several herbs such as *Atractylodes lancea (Thunb.) DC.* [Asteraceae; Atractylodis Rhizoma Alba], *Scrophularia ningpoensis Hemsl.* [Scrophulariaceae; Scrophulariae Radix], *Magnolia officinalis Rehder and E.H.Wilson* [Magnoliaceae; Magnoliae Cortex], and *Prunus armeniaca L.* [Rosaceae; Armeniacae Semen] exert ICCs or nerve mediated prokinetic actions. Moistening and barrier supporting herbs including *Ophiopogon japonicus (Thunb.) Ker Gawl.* [Asparagaceae; Liriopis Tuber], *Angelica gigas Nakai* [Apiaceae; Angelicae Gigantis Radix], and *Paeonia lactiflora Pall.* [Paeoniaceae; Paeoniae Radix Alba] enhance mucus production and attenuate intestinal inflammation. Taken together, these effects on motility, secretion, mucosa, and microbiota provide a coherent mechanistic rationale for the improvements in bowel function with THM in patients receiving opioids. Interestingly, the high frequency herbs identified in this review, such as *Citrus × aurantium f. aurantium* [Rutaceae; Aurantii Immaturus Fructus], *Magnolia officinalis Rehder and E.H.Wilson* [Magnoliaceae; Magnoliae Cortex], *Atractylodes lancea (Thunb.) DC.* [Asteraceae; Atractylodis Rhizoma Alba], *Cannabis sativa L.* [Cannabaceae; Cannabis Semen], and *Rheum officinale Baill.* [Polygonaceae; Rhei Rhizoma] overlapped with those highlighted in a recent systematic review of natural drugs for constipation registered on global clinical trial platforms (Mei et al., 2025). This consistency suggests that herbal prescriptions used for OIC in cancer patients follow a similar pharmacological pattern to that observed in broader constipation research emphasizing prokinetic, secretagogue, and gut-modulating components.

According to the National Comprehensive Cancer Network (NCCN) guidelines, in cancer patients with constipation, lifestyle modifications such as adequate fluid intake and physical activity are implemented as first-line management. The initial pharmacological treatments included stimulant laxatives (such as senna or bisacodyl) and osmotic laxatives (such as polyethylene glycol). In cases of fecal impaction, glycerin or mineral oil enemas are recommended. For persistent constipation, other agents such as lactulose, sorbitol, and magnesium hydroxide may also be considered. In OIC, unresponsive to laxatives with different mechanisms of action, peripherally acting μ-opioid receptor antagonists (PAMORAs) may be considered. However, all these recommendations are graded as category 2A, indicating moderate-level evidence with uniform consensus among experts (National Comprehensive Cancer Network, 2025). Previous studies have shown that osmotic agents such as polyethylene glycol improve stool frequency and consistency compared with stimulant laxatives (Kistemaker et al., 2024), and PAMORAs (e.g., naloxegol or naldemedine) significantly increase spontaneous bowel movements in patients with OIC, including those with cancer (Braun et al., 2024). These data underpin the current guideline recommendations for laxatives and PAMORAs, but also indicate that the existing pharmacologic options provide only partial relief for a substantial proportion of patients. Against this background, the meta-analysis conducted in this study suggests that THM may offer a supportive option showing improvements in OIC-related symptoms. THM should not be regarded as a replacement for guideline-endorsed laxatives or PAMORAs, but rather as a potential adjunctive therapy that could be considered when conventional pharmacologic regimens alone do not provide adequate symptom control or are poorly tolerated. In integrative oncology settings THM might be combined with rehabilitation interventions and acupuncture, which have also shown beneficial effects on constipation and quality of life in oncological patients, to create a multimodal management strategy tailored to individual needs (Chiaramonte et al., 2023). Within such multimodal approaches, safety is a key consideration.

In most studies that reported adverse events, no serious adverse events were associated with THM. Four studies reported mild adverse events, such as diarrhea and nausea; none were severe (Chen et al., 2014; Zhang et al., 2016; Zhu, 2017; Chen et al., 2019). Stimulant and osmotic laxatives used for OIC are known to cause adverse effects such as abdominal cramping and pain, limiting their long-term use (Kistemaker et al., 2024). Magnesium hydroxide can also lead to hypermagnesemia in patients with impaired renal function (Mori et al., 2021). These findings indicate that THM may be a safe alternative therapeutic option for OIC management.

This systematic review and meta-analysis had some limitations. First, the included studies used heterogeneous outcome measures, relying on subjective indicators such as TER, which limited consistent and objective comparisons. Second, the control interventions in the included studies were highly heterogeneous and, in some cases, were not aligned with current clinical guidelines. Phenolphthalein, for instance, has been withdrawn owing to its carcinogenicity. Aloe capsules have been approved by the National Medical Products Administration (NMPA) in China but are not typically recommended for OIC management; furthermore, mosapride citrate is a gastrointestinal prokinetic that is not typically recommended for constipation management. These inconsistencies may have exaggerated the apparent efficacy of THM observed in this review. Third, most of the included RCTs were predominantly conducted in East Asian populations, where THM is deeply embedded in routine care, and cultural familiarity and expectations of benefit may enhance placebo responses and influence symptom reporting. In addition, there was substantial variability in cancer types, disease stages, and anticancer regimens across trials, which may have contributed to between-study heterogeneity and limits the generalizability of these findings to other ethnicities. Besides, the asymmetry of the funnel plot and a positive Egger’s test for TER suggest that small-study and publication bias are likely, particularly in a literature base concentrated in one geographic and cultural context; although trim-and-fill analysis indicated that the direction of the pooled effect remained unchanged after adjustment, the true magnitude of benefit is probably smaller than the unadjusted estimates.

Despite these limitations, this study followed PRISMA guidelines and applied rigorous bias and GRADE assessments. Future high-quality multicenter RCTs using standardized outcomes and comparators are needed to confirm these results and improve their clinical applicability.

## 5 Conclusion

This systematic review and meta-analysis suggest that THM could be considered a promising option for OIC in patients with cancer. THM demonstrated improvements not only in TER but also in constipation-related symptoms, such as stool form, defecation difficulty, and defecation time, with additional benefits for the quality of life. These findings support the clinical potential of THM as a complementary approach for OIC management. Further well-designed, large-scale RCTs with standardized diagnostic criteria and outcome measures are needed to confirm the efficacy and safety of THM in this context.

## Data Availability

The original contributions presented in the study are included in the article/Supplementary Material, further inquiries can be directed to the corresponding author.
