# Supplementary material for: Traditional herbal medicine for opioid-induced constipation in patients with cancer: a systematic review and meta-analysis of randomized controlled trials
Source: Front Pharmacol. 2026 Jan 16;16:1716974. doi: 10.3389/fphar.2025.1716974 (PMC12855494; doi:10.3389/fphar.2025.1716974)
Supplement: Supplementary file 1 [file Supplementaryfile1.docx]

Supplementary Material

# S1. Search terms for each database

1. Pubmed

#1. Search "Neoplasms"[Mesh]

#2. Search (neoplasm* or cancer* or carcino* or malignan* or tumor* or tumour*)

#3. #1 OR #2

#4. Search “Drugs, Chinese Herbal”[MeSH] OR "Plants, Medicinal”[MeSH] OR “Herbal Medicine”[MeSH] OR “Medicine, Kampo”[MeSH] OR "Medicine, Korean Traditional”[MeSH] OR "Medicine, Chinese Traditional”[MeSH]

#5. Search “traditional Korean medicine”[tiab] OR “traditional Chinese medicine”[tiab] OR “traditional oriental medicine”[tiab] OR “Kampo medicine”[tiab] OR herb*[tiab] OR decoction*[tiab] OR botanic*[tiab]

#6. #4 OR #5

#7. Search (“Constipation"[MeSH] OR "Constipation"[TW])

#8. Search ("analgesics, opioid"[MeSH]) OR ("opioid"[TW]) OR ("Opiate"[TW]) OR ("opiate alkaloids"[MeSH]) OR ("analgesics, opioid"[MeSH]) OR ("opiate alkaloids"[MeSH]) OR ("opiates"[TW] OR "opiate alkaloids"[TW]) OR ("analgesics opioid"[TW])

#9. #7 AND #8

#10. Search ("opioid-induced constipation"[MeSH] OR "opioid induced constipation"[TW])

#11. #9 OR #10

#12. #3 AND #6 AND #11

2. Cochrane Library

#1. MeSH descriptor: [Neoplasms] explode all trees

#2. neoplasm* or cancer* or carcino* or malignan* or tumor* or tumour*

#3. #1 OR #2

#4. MeSH descriptor: [Medicine, Kampo] explode all trees

#5. MeSH descriptor: [Medicine, Korean Traditional] explode all trees

#6. MeSH descriptor: [Medicine, Chinese Traditional] explode all trees

#7. #4 OR #5 OR #6

#8. (opioid):ti,ab,kw OR (opiate):ti,ab,kw OR (analgesics):ti,ab,kw (Word variations have been searched)

#9. (constipation):ti,ab,kw OR (gastrointestinal transit):ti,ab,kw OR (bowel dysfunction):ti,ab,kw (Word variations have been searched)

#10 ("opioid-induced bowel dysfunction"):ti,ab,kw OR (opioid-induced constipation):ti,ab,kw (Word variations have been searched)

#11. (#8 AND #9) OR #10

#12. #3 AND #7 AND #11

3. EMBASE

#1 'neoplasms'/exp

#2 neoplasm* OR cancer* OR carcino* OR malignan* OR tumor* OR tumour*

#3 #1 OR #2

#4 'Chinese medicine'/exp

#5 'Korean medicine'/exp

#6 'Kampo medicine'/exp

#7 'juzentaihoto'/exp

#8 #4 OR #5 OR #6 OR #7

#9 ‘opioid induced constipation’/exp

#10 ((('Opioid'/exp) OR ('opiate'/exp)) AND (('constipation'/exp) OR ('Gastrointestinal transit'/exp) OR ('bowel dysfunction'/exp))

#11 #9 AND #10

#12 #3 AND #8 AND #11

4. CNKI

#1 癌 + 肿瘤 + cancer + neoplasm + carcinoma + malignant + tumor

#2 阿片类药物所致便秘 + ((阿片类药物 + 鸦片制剂 + 麻醉性镇痛药) * (便秘 + 肠功能障碍))

#3 ((opioid + opiate) * (constipation + bowel dysfunction)) + opioid-induced constipation

#4 OR/1-3

5. KMBASE

#1 ([KEYWORD=cancer] OR [KEYWORD=neoplasms])

#2 (([ABSTRACT=traditional medicine] OR [ABSTRACT=korean medicine]) OR [ABSTRACT=herb])

6. KISS

#1 cancer AND opioid induced constipation AND Korean medicine

#2 cancer AND opioid induced constipation AND traditional medicine

#3 OR / 1-2

7. NDSL

#1 ABSTRACT=cancer

#2 ABSTRACT=opioid induced constipation

#3 ABSTRACT=Korean medicine | traditional medicine | herb

#4 AND / 1-3

8. CiNii

#1 cancer AND opioid induced constipation AND herbal medicine

#2 cancer AND opioid induced constipation AND traditional medicine

#3 OR / 1-2

# S2. Supplementary Table 1

### Table 1. The ingredients of THM Prescription used in the included studies.

| First Author (Year) | THM Prescription Name | Ingredients of THM Prescription | Dosage (g) |
| --- | --- | --- | --- |
| Chen 2016 | Zengye Chengqi Dec. | *Atractylodes lancea (Thunb.) DC.* [Asteraceae; Atractylodis Rhizoma Alba] | 40 |
|  |  | *Cannabis sativa L.* [Cannabaceae; Cannabis Semen] | 20 |
|  |  | *Trichosanthes kirilowii Maxim.* [Cucurbitaceae; Trichosanthis Fructus] | 20 |
|  |  | *Scrophularia ningpoensis Hemsl.* [Scrophulariaceae; Scrophulariae Radix] | 15 |
|  |  | *Paeonia lactiflora Pall.* [Paeoniaceae; Paeoniae Radix Alba] | 15 |
|  |  | *Angelica gigas Nakai* [Apiaceae; Angelicae Gigantis Radix] | 10 |
|  |  | *Citrus × aurantium f. aurantium* [Rutaceae; Aurantii Immaturus Fructus] | 10 |
|  |  | *Eucommia ulmoides Oliv.* [Eucommiaceae; Eucommiae Cortex] | 10 |
|  |  | *Ophiopogon japonicus (Thunb.) Ker Gawl.* [Asparagaceae; Liriopis Tuber] | 10 |
|  |  | *Rehmannia glutinosa (Gaertn.) Libosch. ex DC.* [Orobanchaceae; Rehmanniae Radix] | 10 |
|  |  | *Natrii sulfas* [inorganic salt; sodium sulfate] | 5 |
| Gao 2010 | Yiqi Runchang Dec. | *Cannabis sativa L.* [Cannabaceae; Cannabis Semen] | 10 |
|  |  | *Codonopsis pilosula (Franch.) Nannf.* [Campanulaceae; Codonopsis Pilosulae Radix] | 10 |
|  |  | *Lycium chinense Mill.* [Solanaceae; Lycii Fructus] | 10 |
|  |  | *Reynoutria multiflora (Thunb.) Moldenke* [Polygonaceae; Polygoni Multiflori Radix ] | 10 |
|  |  | *Rehmannia glutinosa (Gaertn.) Libosch. ex DC.* [Orobanchaceae; Rehmanniae Radix] | 10 |
|  |  | *Scrophularia ningpoensis Hemsl.* [Scrophulariaceae; Scrophulariae Radix] | 10 |
|  |  | *Prunus armeniaca L.* [Rosaceae; Armeniacae Semen] | 7 |
|  |  | *Citrus × aurantium f. aurantium* [Rutaceae; Aurantii Immaturus Fructus] | 5 |
|  |  | *Cistanche deserticola Ma* [Orobanchaceae; Cistanches Herba] | 5 |
|  |  | *Lindera aggregata (Sims) Kosterm.* [Lauraceae; Linderae Radix] | 5 |
| Hou 2017 | Maziren Dec. | *Astragalus mongholicus Bunge* [Fabaceae; Astragali Radix] | 20 |
|  |  | *Cannabis sativa L.* [Cannabaceae; Cannabis Semen] | 15 |
|  |  | *Cistanche deserticola Ma* [Orobanchaceae; Cistanches Herba] | 15 |
|  |  | *Paeonia lactiflora Pall.* [Paeoniaceae; Paeoniae Radix Alba] | 15 |
|  |  | *Prunus persica (L.) Batsch* [Rosaceae; Pruni Armenicae Semen] | 15 |
|  |  | *Citrus × aurantium f. aurantium* [Rutaceae; Aurantii Immaturus Fructus] | 10 |
|  |  | *Angelica gigas Nakai* [Apiaceae; Angelicae Gigantis Radix] | 10 |
|  |  | *Magnolia officinalis Rehder & E.H.Wilson* [Magnoliaceae; Magnoliae Cortex] | 10 |
|  |  | *Rheum officinale Baill.* [Polygonaceae; Rhei Rhizoma] | 10 |
| Li 2012 | Maziren Pill | *Astragalus mongholicus Bunge* [Fabaceae; Astragali Radix] | 30 |
|  |  | *Cannabis sativa L.* [Cannabaceae; Cannabis Semen] | 30 |
|  |  | *Scleromitrion diffusum (Willd.) R.J.Wang* [Rubiaceae; Oldenlandiae Diffusae Herba] | 20 |
|  |  | *Angelica gigas Nakai* [Apiaceae; Angelicae Gigantis Radix] | 15 |
|  |  | *Magnolia officinalis Rehder & E.H.Wilson* [Magnoliaceae; Magnoliae Cortex] | 15 |
|  |  | *Paeonia lactiflora Pall*. [Paeoniaceae; Paeoniae Radix Alba] | 15 |
|  |  | *Citrus × aurantium f. aurantium* [Rutaceae; Aurantii Immaturus Fructus] | 12 |
|  |  | *Curcuma aromatica Salisb.* [Zingiberaceae; Zedoariae Rhizoma] | 12 |
|  |  | *Prunus armeniaca L.* [Rosaceae; Armeniacae Semen] | 10 |
|  |  | *Rheum officinale Baill.* [Polygonaceae; Rhei Rhizoma] | 9 |
|  |  | *Pinellia ternata (Thunb.) Makino* [Araceae; Pinelliae Rhizoma] | 9 |
|  |  | *Glycyrrhiza uralensis Fisch. ex DC.* [Fabaceae; Glycyrrhizae Radix] | 6 |
| Li 2017 | Zhe Sui Gongjie Tang | *Halloysitum rubrum* [mineral; red halloysite clay] | 60 |
|  |  | *Natrii sulfas* [inorganic salt; sodium sulfate] | 15 |
|  |  | *Euphorbia kansui S.L.Liou ex S.B.Ho* [Euphorbiaceae; Euphorbiae Kansui Radix] | 4.5 |
| Liu 2022 | Sini San with Suzi Jiangqi Tang | *Atractylodes lancea (Thunb.) DC.* [Asteraceae; Atractylodis Rhizoma Alba] | 30 |
|  |  | *Crataegus monogyna Jacq.* [Rosaceae; Crataegi Fructus] | 15 |
|  |  | *Raphanus raphanistrum subsp. sativus (L.) Domin* [Brassicaceae; Raphani Semen] | 15 |
|  |  | *Angelica gigas Nakai* [Apiaceae; Angelicae Gigantis Radix] | 10 |
|  |  | *Areca catechu L.* [Arecaceae; Arecae Semen] | 10 |
|  |  | *Prunus armeniaca L.* [Rosaceae; Armeniacae Semen] | 10 |
|  |  | *Citrus × aurantium f. aurantium* [Rutaceae; Aurantii Immaturus Fructus] | 10 |
|  |  | *Magnolia officinalis Rehder & E.H.Wilson* [Magnoliaceae; Magnoliae Cortex] | 10 |
|  |  | *Paeonia lactiflora Pall.* [Paeoniaceae; Paeoniae Radix Alba] | 10 |
|  |  | *Perilla frutescens var. crispa (Thunb.) H.Deane* [Lamiaceae; Perillae Semen] | 10 |
|  |  | *Pinellia ternata (Thunb.) Makino* [Araceae; Pinelliae Rhizoma] | 10 |
|  |  | *Bupleurum chinense DC.* [Apiaceae; Bupleuri Radix] | 6 |
|  |  | *Glycyrrhiza uralensis Fisch. ex DC.* [Fabaceae; Glycyrrhizae Radix] | 3 |
| Peng 2019 | Jiawei Sini Dec. | *Angelica gigas Nakai* [Apiaceae; Angelicae Gigantis Radix] | 15 |
|  |  | *Atractylodes lancea (Thunb.) DC.* [Asteraceae; Atractylodis Rhizoma Alba] | 15 |
|  |  | *Cistanche deserticola Ma* [Orobanchaceae; Cistanches Herba] | 15 |
|  |  | *Aconitum carmichaelii Debeaux* [Ranunculaceae; Aconiti lateralis preparata radix] | 9 |
|  |  | *Astragalus mongholicus Bunge* [Fabaceae; Astragali Radix] | 9 |
|  |  | *Citrus × aurantium f. aurantium* [Rutaceae; Aurantii Immaturus Fructus] | 9 |
|  |  | *Zingiber officinale Roscoe* [Zingiberaceae; Zingiberis Rhizoma] | 9 |
|  |  | *Glycyrrhiza uralensis Fisch. ex DC.* [Fabaceae; Glycyrrhizae Radix] | 6 |
|  |  | *Magnolia officinalis Rehder & E.H.Wilson* [Magnoliaceae; Magnoliae Cortex] | 6 |
| Wei 2020 | Yiqi Zengye Dec. | *Astragalus mongholicus Bunge* [Fabaceae; Astragali Radix] | 50 |
|  |  | *Angelica gigas Nakai* [Apiaceae; Angelicae Gigantis Radix] | 30 |
|  |  | *Atractylodes lancea (Thunb.) DC.* [Asteraceae; Atractylodis Rhizoma Alba] | 30 |
|  |  | *Cannabis sativa L.* [Cannabaceae; Cannabis Semen] | 15 |
|  |  | *Eucommia ulmoides Oliv.* [Eucommiaceae; Eucommiae Corte] | 15 |
|  |  | *Ophiopogon japonicus (Thunb.) Ker Gawl.* [Asparagaceae; Liriopis Tuber] | 15 |
|  |  | *Rehmannia glutinosa (Gaertn.) Libosch. ex DC.* [Orobanchaceae; Rehmanniae Radix] | 15 |
|  |  | *Scrophularia ningpoensis Hemsl.* [Scrophulariaceae; Scrophulariae Radix] | 15 |
|  |  | *Glycyrrhiza uralensis Fisch. ex DC.* [Fabaceae; Glycyrrhizae Radix] | 9 |
|  |  | *Prunus persica (L.) Batsch* [Rosaceae; Pruni Armenicae Semen] | 9 |
| Wu 2018 | Xiaoji Anzhong Dec. | *Astragalus mongholicus Bunge* [Fabaceae; Astragali Radix] | 25 |
|  |  | *Oryza sativa L.* [Poaceae; Oryzae Fructus Germinatus] | 25 |
|  |  | *Ziziphus jujuba Mill.* [Rhamnaceae; Zizyphi Spinosae Semen] | 25 |
|  |  | *Agrimonia eupatoria L.* [Rosaceae; Agrimoniae Herba] | 15 |
|  |  | *Citrus × aurantium f. aurantium* [Rutaceae; Aurantii Immaturus Fructus] | 15 |
|  |  | *Cannabis sativa L.* [Cannabaceae; Cannabis Semen] | 15 |
|  |  | *Paeonia lactiflora Pall.* [Paeoniaceae; Paeoiae Radix Alba] | 15 |
|  |  | *Dendrobium nobile Lindl.* [Orchidaceae; Dendrobii Herba] | 10 |
|  |  | *Hordeum vulgare L.* [Poaceae; Hordei Fructus Germinatus] | 10 |
|  |  | *Rehmannia glutinosa (Gaertn.) Libosch. ex DC.* [Orobanchaceae; Rehmanniae Radix] | 10 |
|  |  | *Prunus armeniaca L.* [Rosaceae; Armeniacae Semen] | 9 |
|  |  | *Prunus persica (L.) Batsch* [Rosaceae; Persicae Semen] | 9 |
| Yi 2018 | Qirong Runchang Liq. | *Angelica gigas Nakai* [Apiaceae; Angelicae Gigantis Radix] | Not clearly reported |
|  |  | *Atractylodes lancea (Thunb.) DC.* [Asteraceae; Atractylodis Rhizoma Alba] |  |
|  |  | *Astragalus mongholicus Bunge* [Fabaceae; Astragali Radix] |  |
|  |  | *Citrus × aurantium f. aurantium* [Rutaceae; Aurantii Immaturus Fructus] |  |
|  |  | *Cannabis sativa L.* [Cannabaceae; Cannabis Semen] |  |
|  |  | *Cistanche deserticola Ma* [Orobanchaceae; Cistanches Herba] |  |
|  |  | *Ophiopogon japonicus (Thunb.) Ker Gawl.* [Asparagaceae; Liriopis Tuber] |  |
|  |  | *Mel* [Apidae; honey] |  |
|  |  | *Morus alba L.* [Moraceae; Mori Fructus] |  |
|  |  | *Polygonatum odoratum (Mill.) Druce* [Asparagaceae; Polygonati Rhizoma] |  |
|  |  | *Prunus persica (L.) Batsch* [Rosaceae; Pruni Armenicae Semen] |  |
|  |  | *Pseudostellaria heterantha var. heterantha* [Caryophyllaceae; Pseudostellaria Maximowicziana] |  |
|  |  | *Rehmannia glutinosa (Gaertn.) Libosch. ex DC.* [Orobanchaceae; Rehmanniae Radix] |  |
|  |  | *Scrophularia ningpoensis Hemsl.* [Scrophulariaceae; Scrophulariae Radix] |  |
|  |  | *Sesamum indicum L.* [Pedaliaceae; Sesami Semen Nigrum] |  |
| Yin 2019 | Zengye Chengqi Dec. | *Scrophularia ningpoensis Hemsl.* [Scrophulariaceae; Scrophulariae Radix] | 30 |
|  |  | *Ophiopogon japonicus (Thunb.) Ker Gawl.* [Asparagaceae; Liriopis Tuber] | 24 |
|  |  | *Rehmannia glutinosa (Gaertn.) Libosch. ex DC.* [Orobanchaceae; Rehmanniae Radix] | 24 |
|  |  | *Rheum officinale Baill.* [Polygonaceae; Rhei Rhizoma] | 9 |
|  |  | *Natrii sulfas* [inorganic salt; sodium sulfate] | 4.5 |
| Zhang 2016 | Yangyin Liqi Dec. | *Scrophularia ningpoensis Hemsl.* [Scrophulariaceae; Scrophulariae Radix] | 24 |
|  |  | *Ophiopogon japonicus (Thunb.) Ker Gawl.* [Asparagaceae; Liriopis Tuber] | 20 |
|  |  | *Rehmannia glutinosa (Gaertn.) Libosch. ex DC.* [Orobanchaceae; Rehmanniae Radix] | 20 |
|  |  | *Cannabis sativa L.* [Cannabaceae; Cannabis Semen] | 18 |
|  |  | *Areca catechu L.* [Arecaceae; Arecae Semen] | 10 |
|  |  | *Codonopsis pilosula (Franch.) Nannf.* [Campanulaceae; Codonopsis Pilosulae Radix] | 10 |
|  |  | *Lindera aggregata (Sims) Kosterm.* [Lauraceae; Linderae Radix] | 9 |
|  |  | *Rheum officinale Baill.* [Polygonaceae; Rhei Rhizoma] | 6 |
|  |  | *Aquilaria sinensis (Lour.) Spreng.* [Thyaeaceae; Aquilariae Resinatum Lignum] | 3 |
|  |  | *Natrii sulfas* [inorganic salt; sodium sulfate] | 3 |
| Zhu 2017 | Tongxia Runchang Dec. | *Atractylodes lancea (Thunb.) DC.* [Asteraceae; Atractylodis Rhizoma Alba] | 60 |
|  |  | *Citrus × aurantium f. aurantium* [Rutaceae; Aurantii Immaturus Fructus] | 30 |
|  |  | *Cannabis sativa L.* [Cannabaceae; Cannabis Semen] | 30 |
|  |  | *Cistanche deserticola Ma* [Orobanchaceae; Cistanches Herba] | 30 |
|  |  | *Angelica gigas Nakai* [Apiaceae; Angelicae Gigantis Radix] | 15 |
|  |  | *Magnolia officinalis Rehder & E.H.Wilson* [Magnoliaceae; Magnoliae Cortex] | 15 |
|  |  | *Prunus armeniaca L.* [Rosaceae; Armeniacae Semen] | 9 |
|  |  | *Actaea racemosa L.* [Ranunculaceae; Cimicifugae Rhizoma] | 6 |
|  |  | *Rheum officinale Baill.* [Polygonaceae; Rhei Rhizoma] | 6 |
| Chen 2014 | Xiaochengqi Dec.  with Zengye Dec. | *Rehmannia glutinosa (Gaertn.) Libosch. ex DC.* [Orobanchaceae; Rehmanniae Radix] | 20 |
|  |  | *Ophiopogon japonicus (Thunb.) Ker Gawl.* [Asparagaceae; Liriopis Tuber] | 15 |
|  |  | *Scrophularia ningpoensis Hemsl.* [Scrophulariaceae; Scrophulariae Radix] | 15 |
|  |  | *Citrus × aurantium f. aurantium* [Rutaceae; Aurantii Immaturus Fructus] | 10-15 |
|  |  | *Magnolia officinalis Rehder & E.H.Wilson* [Magnoliaceae; Magnoliae Cortex] | 10-15 |
|  |  | *Rheum officinale Baill.* [Polygonaceae; Rhei Rhizoma] | 5-10 |
| Li 2013 | Qizhu Zengye Dec. | *Astragalus mongholicus Bunge* [Fabaceae; Astragali Radix] | 30 |
|  |  | *Atractylodes lancea (Thunb.) DC.* [Asteraceae; Atractylodis Rhizoma Alba] | 30 |
|  |  | *Lilium lancifolium Thunb.* [Liliaceae; Lilii Bulbus] | 30 |
|  |  | *Scrophularia ningpoensis Hemsl.* [Scrophulariaceae; Scrophulariae Radix] | 30 |
|  |  | *Trichosanthes kirilowii Maxim.* [Cucurbitaceae; Trichosanthis Fructus] | 30 |
|  |  | *Morus alba L.* [Moraceae; Mori Fructus] | 25 |
|  |  | *Clematis chinensis Osbeck* [Ranunculaceae; Clematidis Radix et Rhizoma] | 15 |
|  |  | *Angelica gigas Nakai* [Apiaceae; Angelicae Gigantis Radix] | 10 |
|  |  | *Citrus × aurantium f. aurantium* [Rutaceae; Aurantii Immaturus Fructus] | 10 |
|  |  | *Ophiopogon japonicus (Thunb.) Ker Gawl.* [Asparagaceae; Liriopis Tuber] | 10 |
|  |  | *Paeonia lactiflora Pall.* [Paeoniaceae; Paeoniae Radix Alba] | 10 |
|  |  | *Polygonatum odoratum (Mill.) Druce* [Asparagaceae; Polygonati Rhizoma] | 10 |
|  |  | *Raphanus raphanistrum subsp. sativus (L.) Domin* [Brassicaceae; Raphani Semen] | 10 |
|  |  | *Rehmannia glutinosa (Gaertn.) Libosch. ex DC.* [Orobanchaceae; Rehmanniae Radix] | 10 |
| Long 2006 | Xin Jia Huang Long Dec. | *Scrophularia ningpoensis Hemsl.* [Scrophulariaceae; Scrophulariae Radix] | 25 |
|  |  | *Ophiopogon japonicus (Thunb.) Ker Gawl.* [Asparagaceae; Liriopis Tuber] | 15 |
|  |  | *Rehmannia glutinosa (Gaertn.) Libosch. ex DC.* [Orobanchaceae; Rehmanniae Radix] | 15 |
|  |  | *Angelica gigas Nakai* [Apiaceae; Angelicae Gigantis Radix] | 10 |
|  |  | *Natrii sulfas* [inorganic salt; sodium sulfate] | 10 |
|  |  | Ginseng Radix | 6 |
|  |  | *Glycyrrhiza uralensis Fisch. ex DC.* [Fabaceae; Glycyrrhizae Radix] | 6 |
|  |  | *Rheum officinale Baill.* [Polygonaceae; Rhei Rhizoma] | 6 |
|  |  | *Stichopus japonicus Selenka* [Stichopodidae; Stichopus] | 2ea |
| Jia 2009 | Jiangni Kuanchang Dec. | *Citrus × aurantium f. aurantium* [Rutaceae; Aurantii Immaturus Fructus] | Not clearly reported |
|  |  | *Galli stomachichum corium* [animal derivative; inner lining of chicken gizzard] |  |
|  |  | *Pentanema britannica (L.) D.Gut.Larr., Santos-Vicente, Anderb., E.Rico & M.M.Mart.Ort.* [Asteraceae; Inulae Flos] |  |
|  |  | *Magnolia officinalis Rehder & E.H.Wilson* [Magnoliaceae; Magnoliae Cortex] |  |
|  |  | *Pinellia ternata (Thunb.) Makino* [Araceae; Pinelliae Rhizoma] |  |
|  |  | *Raphanus raphanistrum subsp. sativus (L.) Domin* [Brassicaceae; Raphani Semen] |  |
|  |  | *Rheum officinale Baill.* [Polygonaceae; Rhei Rhizoma] |  |
|  |  | *Rosa rugosa Thunb.* [Rosaceae; Rosae Rugosae Flos] |  |
| Sun 2015 | Liqi Jiangni Gran. | *Citrus × aurantium f. aurantium* [Rutaceae; Aurantii Immaturus Fructus] | Not clearly reported |
|  |  | *Pentanema britannica (L.) D.Gut.Larr., Santos-Vicente, Anderb., E.Rico & M.M.Mart.Ort.* [Asteraceae; Inulae Flos] |  |
|  |  | *Magnolia officinalis Rehder & E.H.Wilson* [Magnoliaceae; Magnoliae Cortex] |  |
|  |  | *Pinellia ternata (Thunb.) Makino* [Araceae; Pinelliae Rhizoma] |  |
|  |  | *Raphanus raphanistrum subsp. sativus (L.) Domin* [Brassicaceae; Raphani Semen] |  |
| Ma 2016 | Huisheng Sol. | Not reported | |
| Wang 2017 | Fuzheng Runchang  Dec. | *Platycladus orientalis (L.) Franco* [Cupressaceae; Biotae Semen] | 20 |
|  |  | *Ziziphus jujuba Mill.* [Rhamnaceae; Zizyphi Spinosae Semen] | 20 |
|  |  | *Angelica gigas Nakai* [Apiaceae; Angelicae Gigantis Radix] | 15 |
|  |  | *Atractylodes lancea (Thunb.) DC.* [Asteraceae; Atractylodis Rhizoma Alba] | 15 |
|  |  | *Senna tora (L.) Roxb.* [Fabaceae; Cassiae Semen] | 15 |
|  |  | *Codonopsis pilosula (Franch.) Nannf.* [Campanulaceae; Codonopsis Pilosulae Radix] | 15 |
|  |  | *Vachellia farnesiana var. farnesiana* [Fabaceae; Hoelen] | 15 |
|  |  | *Paeonia lactiflora Pall.* [Paeoniaceae; Paeoniae Radix Alba] | 15 |
|  |  | *Rehmannia glutinosa (Gaertn.) Libosch. ex DC.* [Orobanchaceae; Rehmanniae Radix] Preparata | 15 |
|  |  | *Eleutherococcus nodiflorus (Dunn) S.Y.Hu* [Araliaceae; Acanthopanacis cortex] | 10 |
|  |  | *Areca catechu L.* [Arecaceae; Arecae Semen] | 10 |
|  |  | *Prunus armeniaca L.* [Rosaceae; Armeniacae Semen] | 10 |
|  |  | *Citrus × aurantium f. aurantium* [Rutaceae; Aurantii Immaturus Fructus] | 10 |
|  |  | *Citrus × limon (L.) Osbeck* [Rutaceae; Citri Pericarpium] | 10 |
|  |  | *Conioselinum officinale (Makino) K.Ohashi & H.Ohashi* [Apiaceae; Cnidii Rhizoma] | 10 |
|  |  | *Senega officinalis Spach* [Polygalaceae; Polygalae Radix] | 10 |
|  |  | *Wurfbainia compacta (Sol. ex Maton) Škorničk. & A.D.Poulsen* [Zingiberaceae; Amomi fructus] | 9 |
| Chen 2019 | Bianmitong | *Dolomiaea costus (Falc.) Kasana & A.K.Pandey* [Asteraceae; Aucklandiae Radix] | 15 |
|  |  | *Citrus × aurantium f. aurantium* [Rutaceae; Aurantii Immaturus Fructus] | 15 |
|  |  | *Atractylodes lancea (Thunb.) DC.* [Asteraceae; Atractylodis Rhizoma Alba] | 15 |
|  |  | *Vachellia farnesiana var. farnesiana* [Fabaceae; Hoelen] | 15 |
|  |  | *Wurfbainia compacta (Sol. ex Maton) Škorničk. & A.D.Poulsen* [Zingiberaceae; Amomi fructus] | 9 |
|  |  | *Rheum officinale Baill.* [Polygonaceae; Rhei Rhizoma] | 6 |

Dec, decoction; Liq, liquid; Gran, granule; Sol, solution.
